# 

**Published:** 1889-10

**Authors:** 


COMMUNICATIONS.

Facial Neuralgia Consequent upon Pregnancy........................... 473
Diseases of the Mucous Membrane........................................... 476
The Influence of the Various Deposits upon the Teeth and Adjacent Parts........................................................................ 482
Proceedings of the American Dental Association.......................... 485
Georgia State Dental Society................................................... 504
National Association of Dental Faculties.................................... 512
National Association of Dental Examiners.................................. 515

SELECTIONS.

Prof. Mendel's Views of the Therapeutic Value of Hypnotism........ 519

EDITORIAL.

The Dental Protective Association of the United States................. 520
The Seventh Ohio District Dental Society.................................... 521
Ohio Valley Dental Society...................................................... 522
Ohio State Society................................................................ 522
Fifth Annual Meeting............................................................ 523
Buried Gold........................................................................ 523
Board of Dental Examiners' Meeting......................................... 524
For Sale............................................................................ 524

